# Implementation of Electrical Auricular Acupuncture and Low Frequency Modulated Electric Current Therapy in Pain Management of Patients with Knee Osteoarthritis: A Randomized Pilot Trial

**DOI:** 10.3390/jcm8081229

**Published:** 2019-08-15

**Authors:** Irena Krusche-Mandl, Alexandra Kaider, Julia Starlinger, Michael Preschitz, Rupert Schuster, Ronald Kefurt, Peter Marhofer, Maximilian Kasparek, Stefan Hajdu, Sabine Sator-Katzenschlager

**Affiliations:** 1 Department of Orthopedics and Trauma Surgery, Vienna General Hospital, Medical University of Vienna, Waehringer Guertel 18-20, 1090 Vienna, Austria; 2 Center for Medical Statistics, Informatics and Intelligent Systems, Vienna General Hospital, Medical University of Vienna, 1090 Vienna, Austria; 3 Department of Special Anaesthesia and Pain Therapy, Outpatient Pain Center, Vienna General Hospital, Medical University of Vienna, 1090 Vienna, Austria; 4 Department of Surgery, Division of General Surgery, Vienna General Hospital, Medical University of Vienna, 1090 Vienna, Austria; 5 Department of Anaesthesia, General Intensive Care Medicine and Pain Therapy, Vienna General Hospital, Medical University of Vienna, 1090 Vienna, Austria

**Keywords:** knee osteoarthritis, conservative therapy, pharmacological analgesics, electrical auricular acupuncture (EAA), low frequency modulated electric current therapy (LFMECT)

## Abstract

Background: Knee osteoarthritis is a major cause of knee pain. Conservative therapy resources are limited due to adverse effects. Therefore, alternative non-invasive therapy approaches to reduce pain medications are gaining importance. The current study analyses if electrical auricular acupuncture (EAA) or low frequency modulated electric current therapy (LFMECT) could support analgesic treatment. Methods: In a randomized pilot trial patients with painful knee OA were treated with EAA (group 1) or LFMECT (group 2) additional to standard pharmacological analgesic treatment. In total 19 female and 10 male patients with a mean age of 59.1 years (standard deviation ± 13.6) and a mean BMI of 28.9 kg/m^2^ (± 5.2) were included. Patients were randomly assigned to one of the groups stratified for age, gender and BMI. Before starting of the active study period and collecting of the initial data on day 1, all patients received a pharmacological analgesic baseline therapy for one week. At the next study stage patients started their randomly assigned treatment protocol for 42 days and final follow-up was set on day 70. Patients recorded their pain intensity (numerical rating scale; NRS) using a standardized patient diary. The pain free walking time in min was recorded and range of motion was assessed. Results: Rescue medication intake was comparable between both groups on day 42 (*p* = 0.55) and day 70 (*p* = 0.35). After the active study period (day 42) pain scores decreased significantly in both groups (group 1 *p* = 0.02; group 2 *p* = 0.0006). At follow up median pain scores further decreased in group 1 (*p* = 0.0002) and remained at a low level in group 2 (*p* = 0.001). Level of pain decreased in about 50% in both groups and was comparable during the study period. Total mean range of motion (ROM) increased in both groups (group 1 *p* = 0.0003; group 2 *p* = 0.02). Group 1 had more improvement of mean total ROM compared to group 2 (*p* = 0.034). Pain-free walking time increased in both groups and was comparable between both groups (*p* = 0.31). Any adverse effects due to EAA or LFMECT were not observed. Conclusions: Data of the current study indicates that implementation of EAA or LFMECT seems to be beneficial to reduce knee pain and improve knee function in patients with knee osteoarthritis.

## 1. Introduction

Knee osteoarthritis (OA) is a major cause of knee pain. Resources for pain reduction without causing adverse effects are limited and are needed for patients who are not suitable for surgery. Therefore, alternative approaches, such as acupuncture or acupressure, are gaining importance. Classical acupuncture points are located on the “meridians” or on the ears as described by Nogier et al. [1]. Auricular acupuncture points are based on the corresponding somatotopic relation of the ear with different anatomical regions [2]. Stimulation of acupuncture points can be achieved by insertion of fine needles or by electrical- or laser stimulation. The analgesic effect of EAA analgesia seems to be related due to activation of descending inhibitory pain control systems, depression of long-lasting pain-induced patterns in the spinal signal transduction, and release of endogenous opioid peptides, [3,4,5,6]. Stimulation of acupuncture points with EAA had shown to influence local blood supply in the corresponding body region [7].

The P-Stim^®^™ device (Biegler GmbH, Mauerbach, Austria) was developed to provide continuous EAA for several days. It uses special auricular needles, which are placed on selected acupuncture points, and are connected by wires to a microcontroller fixed behind the ear. It applies a defined periodical electrical stimulation followed by stimulation breaks to prevent tolerance or adaption. In patients with chronic cervical or low back pain electrical auricular acupuncture (EAA) using the P-Stim^®^™ device showed to be more effective than conventional auricular needle acupuncture [7,8,9].

**Figure 1:**
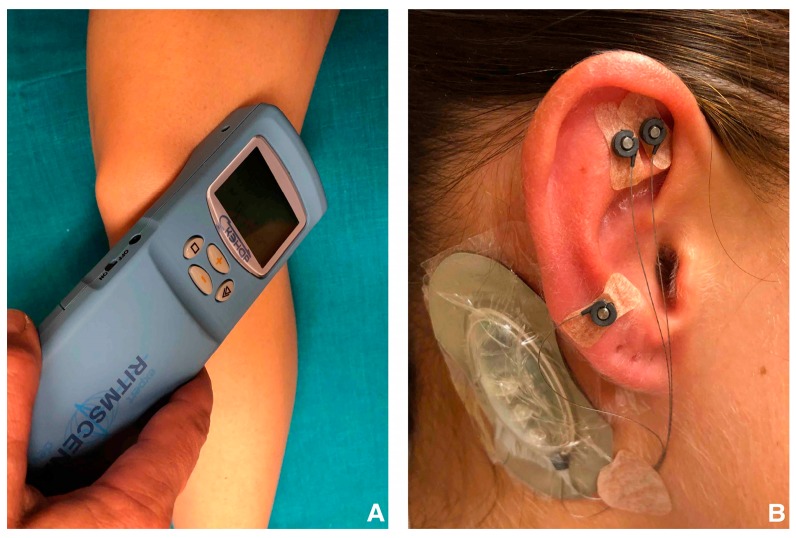
The EAA and LFMECT devices are presented. (**A**) Low frequency modulated electric current therapy (LFMECT) was performed using the SCENAR^®^™ device. The body acupuncture-meridians, bladder-meridian and Bachmann-points were stimulated on an outpatient basis. (**B**) For electrical auricular acupuncture (EAA) the P-Stim^®^™ device was used. Before insertion of the acupuncture needles, skin resistance was measured with an electrical conductance meter to identify the exact acupuncture points (Shen Men 55, knee 49, and cushion 29). Titan disposable auricular needles were inserted at the dominant side and connected to the P-Stim^®^™ device. Patients received continuous low-frequency EAA for 96 hours on an outpatient basis.

Another non-invasive treatment modality is low frequency modulated electric current therapy (LFMECT) with the self-controlled energo-neuro adaptive regulation device (SCENAR^®^™, Enerbalance GMBH, Vienna, Austria) (Figure 1), which is a handheld electrotherapeutic device to stimulate acupuncture-points. The SCENAR^®^™ devices utilizes innervation of C-fibers in the neural system to trigger a release of neuro-regulative-peptides to stimulate pain relief [10]. In comparison to other electrostimulation systems, the SCENAR^®^™ is able to measure the patient’s individual response to the electrostimulation and it accordingly modifies the upcoming impulses.

The number of randomized controlled trials investigating needle acupuncture for knee osteoarthritis (OA) has increased in the last years [11,12]. In contrast limited data about EAA and LFMECT are available. Therefore, the current study analyses if electrostimulation of acupuncture points can support pain management in patients with knee OA.

## 2. Material and Methods

The current randomized pilot trial assessed patients with painful knee OA undergoing EAA or LFMECT additional to standard pharmacological analgesic treatment. The study was approved by the Institutional Review Board on the 5 April 2007 (EK 097/2007) and written informed consent was obtained from all participants.

**Figure 2:**
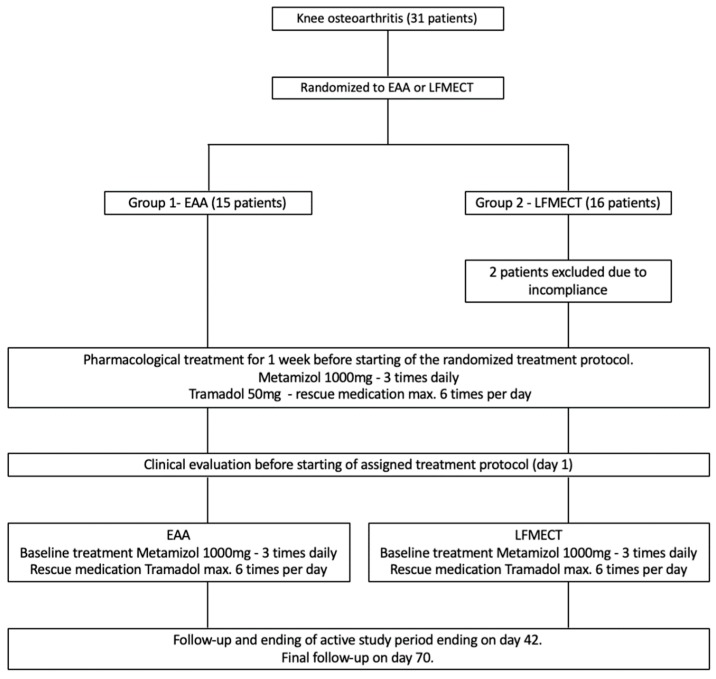
Flowchart showing the study protocol.

Patients were randomly assigned to two groups (Figure 2). In group 1 patients underwent EAA and in group 2 LFMECT additional to standard pharmacological analgesic treatment. All patients were treated at a specialized outpatient pain centre. Before beginning of their randomly assigned treatment protocol, patients received a standardized baseline oral pharmacological analgesic treatment for one week.

Inclusion criteria were age of 18 years or older, a history of knee pain of at least three months, knee pain on most of the days of the past month, an average pain level of ≥3 out of 10 on a numerical rating scale (NRS 0 = no pain, 10 = maximal pain), knee stiffness in the morning of maximal 30 min duration and radiological knee OA (grade >1 according to Kellgren and Lawrence [13]). Exclusion criteria were pregnancy, neurological or psychiatric disorders, pacemaker, cardiac malformation, valvular transplant (risk of endocarditis), acute infection, chronic infection disease (HIV, hepatitis) and immunosuppression (corticoid therapy). Additionally, patients with contraindications for vagal stimulation (e.g., bronchial asthma) were excluded.

Further exclusion criteria were coagulopathy, contraindication for Metamizol or Tramadol intake, skin disorders (ear or knee) and pre-existing pain medication intake (≥ grade II world health organization (WHO)).

In total 31 patients were recruited at the outpatient pain centre. Patients were randomly assigned to one of the intervention groups (EAA or LFMECT) stratified for age, gender, and BMI. Randomization was performed as previously reported by Sator-Katzenschalger et al. using computer-generated randomization [8]. Patients were blinded to the treatment protocol of the other study arm. EAA or LFMECT were performed by specialized study nurses and the examiners were blinded for the patient’s treatment protocol.

15 patients were randomized to the EAA group (group 1) and 16 patients to the LFMECT group (group 2). Two female patients in the LFMECT (group 2) were excluded due to incompliance. Finally, 19 female and 10 male patients with a mean age of 59.1 years (± 13.6) and a mean BMI of 28.9 kg/m^2^ (± 5.2) were included.

Before beginning of their assigned treatment protocol, patients received a pharmacological oral analgesic baseline treatment (Metamizol 1000 mg, 3 times daily and rescue medication with up to six times, 50 mg tramadol per day) for one week. After one-week patients were clinically evaluated and baseline data were collected. In the next study stage, all patients started their assigned treatment protocol (EAA or LFMECT). This time point was set as day 1. The active study period was six weeks (42 days). All patients received EAA or LFMECT on day 1, 7, 14, 21, 28, and 35. All clinical assessments were performed by three fourth year medical students, who were supervised by seven physicians at the outpatient pain center in the midmorning. The active study period ended on day 42 and final follow-up was set on day 70. Patients recorded their required daily rescue medication intake, pain intensity (numerical rating scale; NRS), psychological well-being, quality of sleep, nausea, emesis, and fatigue in a standardized patient diary. Clinical evaluation included knee effusion and active range of motion (ROM). All examiners were trained by an experienced orthopedic trauma surgeon and they trained on several patients with OA not related to the current study. Knee effusion was assessed with the patellar tap test and was rated as positive or negative. ROM was measured according to the neutral zero method with a goniometer using the greater trochanter, lateral femoral epicondyle, fibular head, and lateral malleolus as reference points. All measurements were performed three times and the means were recorded. Moreover, the pain free walking time in min was recorded under standardized conditions. Patients were asked to walk on a flat walking track of 400m length with a consistent walking speed of 3 km/h until they were suffering knee pain.

In group 1 patients received additional to the standardized basic pharmacological analgesic therapy (Metamizol 1000 mg, three times daily and rescue medication with up to six times, 50 mg tramadol per day) electrical auricular acupuncture (EAA) using the P-Stim^®^™ device (Figure 1). Before insertion of the acupuncture needles, skin resistance was measured with an electrical conductance meter (multipoint selection pen^®^™, Biegler GmbH, Mauerbach, Austria) to identify the acupuncture points (Shen Men 55, knee 49, and cushion 29). Titan disposable auricular needles (27 gauge, 3 mm length; Biegler GmbH, Mauerbach, Austria) were inserted in the acupuncture points at the dominant side and connected to the P-Stim^®^™ device. Patients in the auricular acupuncture group (group 1) received continuous low frequency EAA for 96 hours on an outpatient basis. The P-Stim^®^™ device and acupuncture needles were withdrawn by the patients 96 hours after insertion.

Participants in group 2 had in addition to the standardized basic pharmacological treatment low frequency modulated electric current therapy (LFMECT) using the SCENAR^®^™ device (Figure 1). It is a handheld electrotherapeutic device with two integrated electrodes. One electrode delivers computer-modulated electrostimulation via the patient’s skin, while the other one monitors the skin impedance constantly, thus providing a biofeedback mechanism. The SCENAR^®^™ impulse is carried by afferent nerve pathways to the regulatory centers in the brain and triggers a respond signal of the efferent nerves. The LFMECT device SCENAR^®^™ analyze the nerve response and adapts the next impulse for each patient individually. The pathway of pain relief of this technique utilizes the stimulation of the C-fiber neural system. A sufficiently stimulation of this system triggers the release of neuro-regulative-peptides which results in pain relief, (Ing 2007). In the current study following acupuncture points were stimulated with the SCENAR^®^™ device: the body acupuncture meridians, bladder meridian, and Bachmann points.

### Statistical Analysis

The primary outcome parameter was required daily intake of rescue medication. Comparisons between the two groups were performed using the nonparametric Wilcoxon rank sum test. Secondary outcome parameters were pain (NRS), ROM, and pain-free walking time. Continuous outcome variables are presented by mean (± standard deviation). For variables with skewed distribution the median (quartiles) was used. Due to the skewed distribution log-transformed values of the pain-free walking time were used. Analyses of covariance (ANCOVA) models were used for comparison of the outcome variables pain, ROM and pain-free walking time, between the two intervention groups, including the respective baseline values (day 1) as a covariate. Repeated measures analysis of variance (ANOVA) models were calculated to evaluate the secondary outcome variables pain and psychological well-being, compared to baseline values, additionally including follow-up values (day 70). The nonparametric Wilcoxon signed rank test was used to evaluate changes in the subjective outcome variable sleep, as it was not normally distributed. All statistical tests were two-sided and a *p* value < 0.05 was considered statistically significant.

## 3. Results

**Figure 3:**
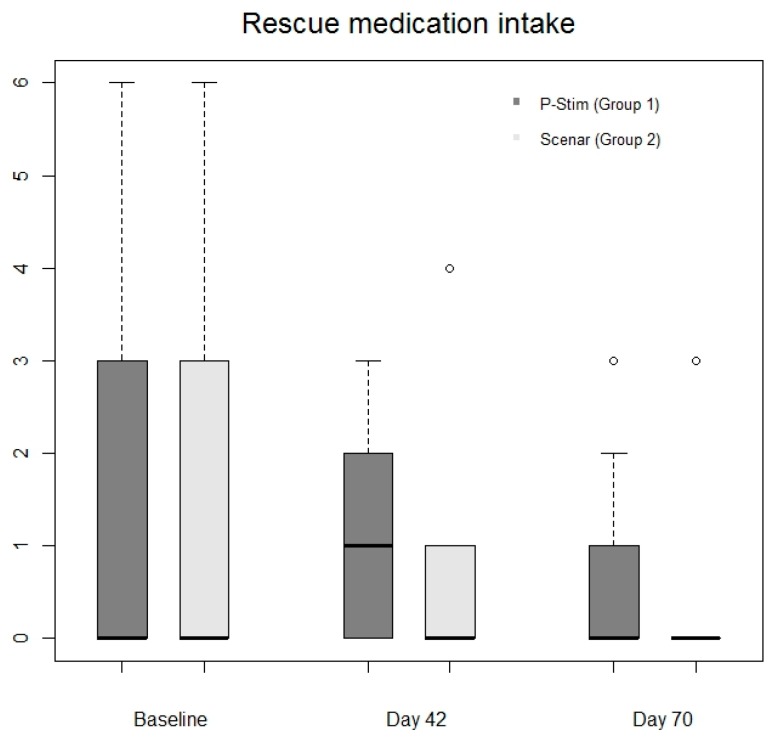
Comparison of required rescues medication.

**Figure 4:**
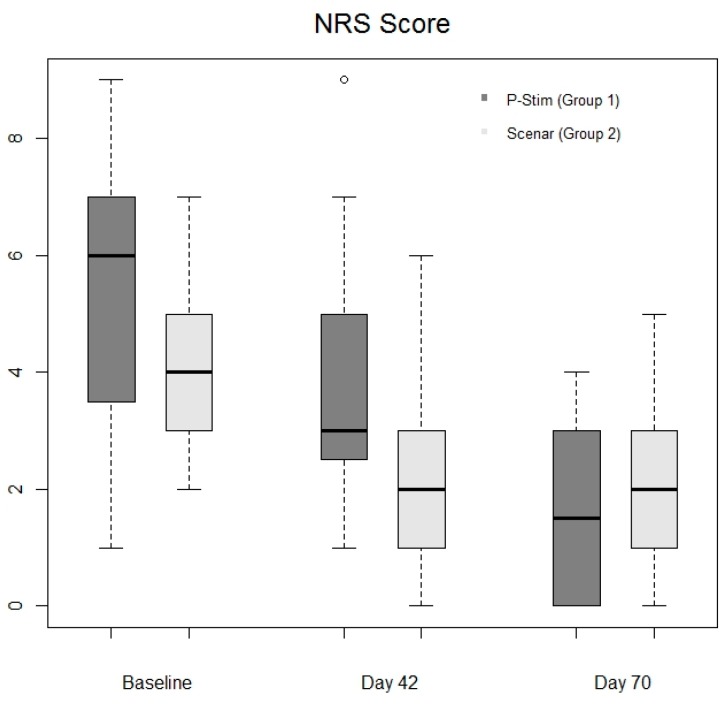
Comparison of pain intensity measured by numerical rating scale (NRS).

Rescue medication intake was comparable in both groups on day 42 (*p* = 0.55) and day 70 (*p* = 0.35) (Table 1 and Figure 3). Pain scores after baseline therapy were comparable between the groups (day 1 in group 1 (6 (Q1 = 3; Q3 = 7)) and group 2 (4 (Q1 = 3; Q3 = 5)). After the active study period (day 42) pain scores decreased significantly in both groups (group 1: 3 (2;5), *p* = 0.02; group 2: 2 (1;3), *p* = 0.0006). At follow up median pain scores further decreased in group 1 (median 1.5 (0;3) *p* = 0.0002) and remained at a low level in group 2 (median 2 (1;3), *p* = 0.001). Level of pain decreased in both groups in about 50% (Figure 4) and pain scores were comparable during the whole study period (Figure 4).

On day 1 mean total ROM was in group 1 105° (± 21) and in group 2 101° (± 28). At the end of the treatment mean total ROM increased in both groups (group 1 126° (± 12), *p* = 0.0003; group 2 114° (± 19), *p* = 0.02). Group 1 showed more improvement of mean total ROM compared to group 2 (*p* = 0.034). The mean extension lag in group 1 decreased from 6° (± 10) at baseline assessment to 2° (± 4) at the end of treatment. In group 2 the mean extension lag improved from 3° (± 5) to 1° (± 3).

Pain-free walking time was after baseline therapy 30 min (10;45) in group 1 and 20 min (10;60) in group 2. At the end of the active study phase an increased pain-free walking time was recorded in both groups (group 1, 90 min (60;180), *p* = 0.0001, group 2 60 min (40;100), *p* = 0.0006). The improvement of the pain-free walking time was comparable (*p* = 0.31). Effusion was extremely rarely observed despite an increasing activity level during the study period.

Psychological well-being and sleep were analyzed together as the results were comparable. Psychological well-being improved in both groups during the active study period (*p* = 0.005) and improved further at final follow-up compared to baseline values (*p* < 0.0001). Also, an improvement of psychological well-being between the end of the active study period and final follow-up (*p* = 0.02) was observed in both groups (Table 2).

Quality of sleep was comparable between the beginning and the end of the active study period (*p* = 0.07). Quality of sleep improved in both groups at final follow-up (*p* = 0.03) (Table 2).

Occurrence of nausea, emesis, and fatigue was caused mainly due to side effects of tramadol intake and was were analyzed together. Nausea was rarely reported during baseline therapy and occurrence of emesis was not recorded during the whole study period. Fatigue values were decreased during the study period and stayed at a low level at final follow-up.

Adverse effects of EAA such as occurrence of auricular hematoma, local infection, or hypotension did not occur. Also, patients undergoing LFMECT did not report any adverse events.

## 4. Discussion

Implementation of EAA or LMFECT seems to be beneficial to support pain management in knee OA. Data of the current study indicates that these therapy modalities seems to be beneficial to reduce knee pain and improve knee function in patients with knee OA.

Acupuncture for patients with OA or chronic pain seems to be a valuable additional treatment option for pain reduction and functional improvement [14,15,16]. The aging population and increasing incidence of obesity are increasing the risk of knee osteoarthritis [17]. Therefore, conservative therapy modalities are gaining in importance, in particular for patients who are at high risk for perioperative and postoperative complications in total joint replacement surgery. For these patients EAA and LFMECT might be encouraging treatment options. Moreover, in these patients a decreased amount of pharmacological analgesics would be beneficial.

Mavrommatis et al. [18] compared in a randomized, placebo-controlled trial three different treatment protocols for patients with knee OA. Group 1 additionally underwent pharmacological treatment acupuncture, group 2 sham acupuncture (needling at defined non-acupuncture points), and group 3 had stand-alone pharmacological treatment. They revealed that acupuncture, as an add-on therapy was more effective than sham acupuncture and stand-alone pharmacological treatment. Scharf et al. [19] performed a three-armed randomized investigation comparing traditional Chinese acupuncture, sham acupuncture, and pharmacological analgesics in patients with chronic pain due to knee OA. In comparison to the study by Mavrommatis et al. [18], Scharf et al. [19] revealed that acupuncture and also sham acupuncture resulted in a higher clinical improvement compared to pharmacological analgesics. They suggest that these results might be a result of an increased patient attention by the practitioners or a placebo effect due to acupuncture and needling. Hinman et al. [20], conducted a prospective randomized study and reported modest improvement in pain comparing acupuncture versus non-acupuncture in patients with chronic knee pain due to osteoarthritis. In contrast to the current study, acupuncture was performed as a stand-alone therapy, whereas in the current study EAA and LFMECT were used as an add-on therapy additionally to standard pharmacological treatment. The current study revealed that EAA and LFMECT support the reduction of required pharmacological medications. Moreover, data of the current study indicates that EAA and LFMECT have a continuing beneficial effect on psychological well-being and a reduction of pain beyond the active study period.

Nonetheless following limitations of the current study have to be considered: (1) Only a limited number of patients were analyzed, however, patients were prospectively and randomly allocated to the intervention groups. (2) An intra- and interobserver reliability analysis of evaluation of knee effusion and ROM measurement was not performed prior the study. (3) Patients were not controlled for level of physical activity, daily working hours or weight changes. (4) The pain free walking time test is not validated for knee osteoarthritis in the literature, however all measurement in the current study were performed under standardized, controlled and reproducible conditions. (5) A control group only receiving pharmacological analgesic treatment was not included. Further randomized trials comparing EAA and LFMECT with patients receiving stand-alone pharmacological analgesic treatment are needed.

## 5. Conclusions

The current study indicates that implementation of EAA and LFMECT in conservative pain management of patients with knee osteoarthritis seems to be beneficial to reduce knee pain and improve knee function. Moreover, EAA and LFMECT showed a continuing effect and might be an additional treatment modality in particular for patients with severe medical comorbidities who are at high risk for complications in total joint replacement surgery.

## Figures and Tables

**Table 1:** Required daily pharmacological rescue medication intake after baseline therapy, at the end of the active study period and at follow-up of all groups.

Parameter	Group 1	Group 2
Group	EAA	LFMECT
Gender (female/male)	10/5	9/5
Age	56.5 (± 13.4)	61.8 (± 13.7)
BMI	29.8 (± 5.6)	27.9(± 4.8)
Rescue medication after baseline therapy (day 1) *	0 (0;3)	0 (0;3)
Rescue medication (day 42) *	1 (0;2)	0 (0;1)
Rescue medication (day 70) *	0 (0;1)	0 (0;0)

EAA, electrical auricular acupuncture; LFMECT, low frequency modulated electric current therapy; Variables are presented by mean (± standard deviation) or median (quartiles 25%–75%); * Median number of 50 mg tramadol intakes per day.

**Table 2:** Pain scores, psychological well-being and quality of sleep after baseline therapy, at the end of the active study period and at follow-up.

Parameter	Group 1	Group 2
Pain score after baseline therapy (day 1)	6 (3;7)	4 (3;5)
Pain score end of treatment (day 42)	3 (2;5)	2 (1;3)
Pain score at follow-up (day 70)	1.5 (0;3)	2 (1;3)
Psychological well-being after baseline therapy (day 1)	5 (2;6)	2 (1;4)
Psychological well-being at the end of therapy (day 42)	3 (2;4)	2 (1;3)
Psychological well-being at follow-up (day 70)	1.5 (0.5;3)	0 (0;2)
Quality of sleep after baseline therapy (day 1)	1 (0;2)	1 (0;2)
Quality of sleep at the end of treatment (day 42)	0 (0;2)	0 (0;1)
Quality of sleep at follow-up (day 70)	0 (0;1)	0 (0;0)

Variables are presented by median (quartiles 25%–75%).
